# AutoEpiCollect 2.0: A Web-Based Machine Learning Tool for Personalized Peptide Cancer Vaccine Design

**DOI:** 10.3390/molecules30244702

**Published:** 2025-12-08

**Authors:** Clifford A. Kim, Nina Shelton, Madhav Samudrala, Kush Savsani, Sivanesan Dakshanamurthy

**Affiliations:** 1 Department of Oncology, Lombardi Comprehensive Cancer Center, Georgetown University School of Medicine, Washington, DC 20007, USA; 2 College of Arts and Sciences, The University of Virginia, Charlottesville, VA 22903, USA; 3 Department of Surgery, Virginia Commonwealth University, Richmond, VA 23219, USA

**Keywords:** personalized cancer vaccine, peptide vaccine, cervical squamous cell carcinoma, cervical intraepithelial neoplasm, human papillomavirus, breast carcinoma, triple negative breast cancer, machine learning, RNA sequencing

## Abstract

Personalized cancer vaccines are a key strategy for training the immune system to recognize and respond to tumor-specific antigens. Our earlier software release, AutoEpiCollect 1.0, was designed to accelerate the vaccine design process, but the identification of tumor-specific genetic variants remains a manual process and is highly burdensome. In this study, we introduce AutoEpiCollect 2.0, an improved version with integrated genetic analysis capabilities that automate the identification and prioritization of tumorigenic variants from individual tumor samples. AutoEpiCollect 2.0 connects with RNA sequencing and cross-references the resulting RNAseq data for efficient determination of cancer-specific and prognostic gene variants. Using AutoEpiCollect 2.0, we conducted two case studies to design personalized peptide vaccines for two distinct cancer types: cervical squamous cell carcinoma and breast carcinoma. Case 1 analyzed five cervical tumor samples from different stages, ranging from CIN1 to cervical cancer stage IIB. CIN3 was selected for detailed analysis due to its pre-invasive status and clinical relevance, as it is the earliest stage where patients typically present symptoms. Case 2 examined five breast tumor samples, including HER2-negative, ER-positive, PR-positive, and triple-negative subtypes. In three of these breast samples, the same epitope was identified and was synthesized by identical gene variants. This finding suggests the presence of shared antigenic targets across subtypes. We identified the top MHC class I and class II epitopes for both cancer types. In cervical carcinoma, the most immunogenic epitopes were found in proteins expressed by HSPG2 and MUC5AC. In breast carcinoma, epitopes with the highest potential were derived from proteins expressed by BRCA2 and AHNAK2. These epitopes were further validated through pMHC-TCR modeling analysis. Despite differences in cancer type and tumor subtype, both case studies successfully identified high-potential epitopes suitable for personalized vaccine design. The integration of AutoEpiCollect 2.0 streamlined the variant analysis workflow and reduced the time required to identify key tumor antigens. This study demonstrates the value of automated data integration in genomic analysis for cancer vaccine development. Furthermore, by applying RNAseq in a standardized workflow, the approach enables both patient-specific and population-level vaccine design, based on statistically frequent gene variants observed across tumor datasets. AutoEpiCollect 2.0 is freely available as a website based tool for user to design vaccine.

## 1. Introduction

Cervical squamous cell carcinoma (CSCC) is correlated to high-risk HPV strains such as HPV-16 and HPV-18 which lead to persistent high-risk human papillomavirus (HPV) infections that cause cancer to develop in the ectocervix near the squamocolumnar junction [1]. The introduction of prophylactic HPV vaccines, commonly known as Gardasil 9, has shown promise in reducing the prevalence of HPV infections and related precancerous lesions. Gardasil 9 is a recombinant vaccine that contains virus-like particles resembling the outer shell of nine different HPV strains but does not contain any viral DNA [2]. Yet, cervical cancer remains a significant global public health challenge, with it being the fourth most common cancer in women globally [3]. In low- and middle-income countries where access to screening and medical therapies may be limited, the burden of disease remains high. Therefore, there is an ongoing need for the development of effective therapeutic agents for patients who develop invasive disease such as personalized peptide cancer vaccines. In a study by Panahi et al., multiepitope peptide cancer vaccines were designed for a murine model from four types of high-risk HPVs using bioinformatics tools. Both prophylactic and therapeutic scenarios were studied and were successful in the complete rejection of tumors within mice [4]. Further advancement of this study requires analysis and assessment of applicability in human models.

Another cancer that is prevalent amongst women is breast carcinoma (BC). BC can be categorized by the presence of specific hormone receptors, with triple-negative breast cancer (TNBC) lacking expression of estrogen receptors (ER), progesterone receptors (PR), and human epidermal growth factor receptor 2 (HER2) [4]. TNBC remains the most aggressive form of BC and the most challenging to treat. Cancer staging, tumor genetic profiles, and patient premenopausal or postmenopausal status largely contribute to prognosis and treatment options [4]. Ultrasounds and mammograms serve as the mainstay for identifying suspicious masses, with tissue biopsies used to confirm the diagnosis. The 5-year survival rate for BC is between 93 and 98%, dependent on patient demographics (e.g., race, ethnicity, age, socioeconomic status, etc.) and tumor morphological properties (e.g., tumor size, local or regional invasion, hormone receptor status, etc.), among other factors.

Clinical standards of care for BC are generally wide and dependent on breast cancer type, but may include neoadjuvant chemotherapy to shrink the tumor before lumpectomy or mastectomy [4]. Treatment depends on the specific staging and classification and may include agents such as selective estrogen receptor modulators (SERMs), selective estrogen receptor downregulators (SERDs), aromatase inhibitors (AIs), etc. There are currently no vaccines used as first-line therapy for the treatment of breast cancer. Still, there is interest in targeting tumor-associated genes to elicit an immune response specific to breast tumor cells [5]. In addition, ongoing clinical trials aim to identify new target proteins on cancer cells to treat and improve patient outcomes. Immunotherapies, especially checkpoint inhibitors like pembrolizumab, have shown promise by producing lasting responses in advanced disease [6]. Additionally, recent advancements in molecular biology and genomics have resulted in high-throughput sequencing technologies, enabling the identification of key genetic alterations and pathways involved in carcinogenesis. The product of such sequence technologies offers potential targets for personalized therapeutic interventions. Our study incorporates human samples of CSCC and BC to demonstrate the highly effective genetic analysis and epitope optimization capabilities of AutoEpiCollect 2.0.

Personalized peptide vaccine design involves sequencing RNA from tumor samples and identification of somatic mutations that act as highly specific targets for an individual’s cancer. Studies have shown that high-throughput sequencing technologies have directly influenced advancements seen in personalized peptide vaccine development [7,8,9]. In our previous study using IntegralVac, an integrated machine learning framework, we demonstrated rapid, accurate, and scalable identification of immunogenic, major histocompatibility complex-binding (MHC-binding) epitopes against evolving pathogens and cancer antigens. This was achieved through its native DeepVacPred, MHCSeqNet, and HemoPI capabilities [10]. We then developed machine learning models for the in silico prediction of non-toxic, non-allergenic epitopes capable of activating tumor-infiltrating lymphocytes, namely AutoEpiCollect and AutoPepVax [11,12]. Using these models, we designed pan-cancer vaccines targeting missense mutations commonly found across several cancer types. However, traditional peptide vaccine development requires the practical identification of carcinogenic variants that create neoantigens. In addition, peptide vaccine development is extremely time-consuming due to the meticulous gene analysis needed to identify these variants [7]. While AutoEpiCollect automates the collection of physicochemical properties and immunological data, there remains an opportunity to improve genetic variant analysis. In this study, we integrate the key findings from our previous studies and introduce an unprecedented approach by developing an end-to-end automated vaccine design process using AutoEpiCollect 2.0. AutoEpiCollect 2.0 integrates Partek Flow, a next generation RNA sequencing tool, and automates the manual analysis of genetic variants from a specified cancer sample involved in peptide cancer vaccine design. This analysis generates a list of ranked epitopes used for designing an effective personalized peptide cancer vaccine.

Our primary goal in this study is to demonstrate the flexibility of the personalized vaccine development process using two methods: peptide vaccine development through the identification of generalized variants known to cause cancer (module 1) and peptide vaccine development through cancer-specific variants (module 2). The generalized variants refers to variants that are known to cause nonspecific cancer whereas cancer-specific variants refers to variants that have an established association to a specific cancer. Furthermore, we achieved a secondary goal of improved latency times in the previously manual genetic analysis process by automating this in AutoEpiCollect 2.0. We used AutoEpiCollect 2.0 to design peptide vaccines for a CSCC case designated as case 1 and a BC case designated as case 2. Case 1 uses module 1, while case 2 follows module 2 as outlined above. In addition, we study the future implications that personalized cancer vaccines provide for a generalized population vaccine as well as a vaccine designed to address individuals who are clinically healthy but have high-risk gene variants.

## 2. Results

### 2.1. AutoEpiCollect 2.0 User Interface

**Figure 1:**
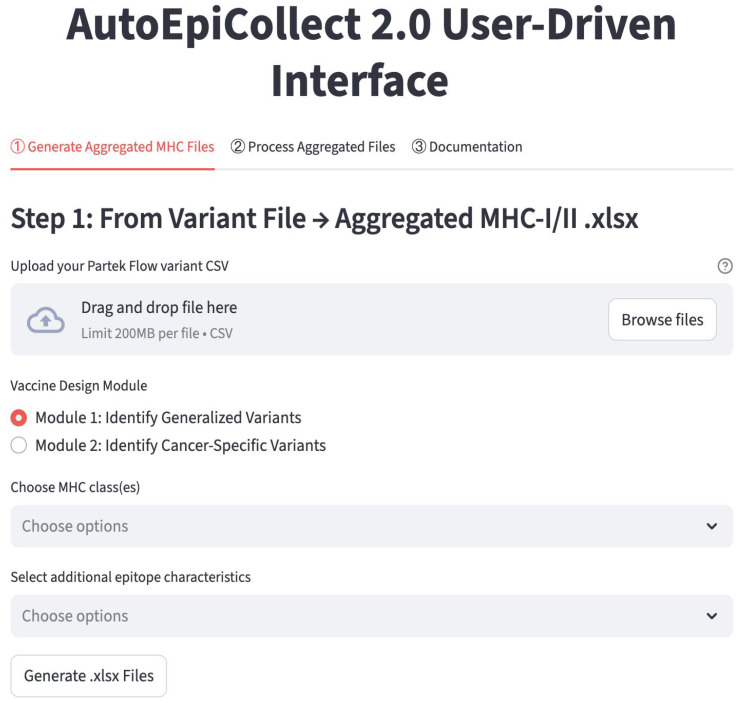
The “Personalized” Page from the AutoEpiCollect 2.0 Website. The personalized cancer vaccine process is carried out in 2 distinct steps. The first step uses the gene and mutation data from Partek Flow to output Excel files containing collected epitopes. Along with the Excel files are FASTA-formatted files with a unique set of peptides for user antigenicity collection through VaxiJen. In the second step, the user must input the antigenicity data along with the Excel data to obtain the final personalized cancer vaccine composition. Detailed information about using AutoEpiCollect 2.0 is located in the “Documentation” tab, as seen in the figure.

AutoEpiCollect 2.0 operates through a straightforward two-step workflow that allows users to move from raw variant data to fully ranked and filtered major histocompatibility complex (MHC) class I and class II epitope candidates. An image of the personalized cancer vaccine section of the website is shown in Figure 1. In the first step, “Generate Aggregated MHC Files,” users upload the .csv variant file generated by Partek Flow, and the software automatically filters for missense mutations occurring within coding exons while removing nonsense mutations and organizing the remaining variants by gene and mutation frequency. Users then select one of two modules, described in Section 4.2, that further reduces the number of genes. Module 1 is a generalized module that retains genes with carcinogenic or prognostic relevance based on the Human Protein Atlas, and module 2 is a cancer-specific module that cross-references the user’s chosen cancer subtype with the top mutated genes in Catalogue of Somatic Mutations in Cancer (COSMIC). After choosing whether to analyze MHC class I epitopes, class II epitopes, or both, and selecting any additional epitope characteristics they want AutoEpiCollect 2.0 to collect, the software generates all possible peptides surrounding each mutation and gathers the relevant immunological and physicochemical characteristics. At the end of this first step, the user downloads a .zip file containing an aggregated Excel spreadsheet of all predicted epitopes along with a FASTA file listing the unique peptide sequences. Because VaxiJen now uses a reCAPTCHA system that blocks automated web scraping, the user must upload this FASTA file to VaxiJen manually to obtain antigenicity values before proceeding. More specific directions on how to use VaxiJen and which files to upload are in the “Documentation” tab (Figure 1).

In the second step, “Process Aggregated Files,” users upload both the aggregated epitope spreadsheets from Step 1 and the antigenicity .txt output from VaxiJen. AutoEpiCollect 2.0 merges the datasets, identifies which additional characteristics are present, and allows users to choose whether to apply the machine learning-based epitope scoring algorithm, perform further filtering based on other epitope characteristics, or calculate population coverage. When scoring is selected, the software normalizes all features and applies the probabilistic logistic regression model developed during AutoEpiCollect 1.0 to assign each epitope a “potential” value that reflects its likelihood of functioning as an effective immunogenic candidate. Users may then filter epitopes based on half-life, instability index, toxicity, or predicted IFN-γ release, and, if desired, generate population-coverage estimates for both class I and class II epitopes. After processing is complete, AutoEpiCollect 2.0 provides a final .zip file containing the fully merged, ranked, filtered, and, when requested, population-coverage-optimized epitope outputs. This two-step structure allows users to complete the personalized vaccine workflow without requiring prior bioinformatics experience. Detailed instructions on how to navigate the website and use AutoEpiCollect 2.0 are available directly on the site at https://autoepicollect2.streamlit.app/ (accessed on 2 December 2025).

### 2.2. Identifying Gene Variants and Analyzing RNAseq Data

We performed RNA sequencing (RNAseq) analysis on the data for each tumor sample using the hg38 human reference genome in Partek Flow. Our analysis identified and annotated the genetic variants found in each sample. In case 1, 1,233,626 mutations were identified across all five samples. Epitopes from all samples were examined, but we selected the cervical intraepithelial neoplasm grade 3 (CIN3) sample to serve as a proof of concept in this study. These mutations were then filtered to retain exons and exclude nonsense mutations, resulting in a dataset comprising 5387 exons with single-nucleotide polymorphism (SNPs) and their associated genes. The PartekFlow RNAseq output files for the control, CIN1, CIN3, stage IA1, and stage IIB samples in case 1 can be found in Supplementary Materials Files S14, S15, S16, S17, and S18, respectively.

In cases 1 and 2, AutoEpiCollect 2.0 extracted and filtered reads to include SNPs within exons, leading to a missense mutation in the RNA coding region. Each selected tumor and non-tumor samples were filtered to the final number of variants of interest (Table 1). The total number of reads encompassed the entire genome compared to the RNAseq analysis and showed the total count of raw DNA sequencing. Full-spectrum genome analysis, which is the entire procedure outlined in Section 4.1, was performed using the next-generation sequencing (NGS) capabilities of Partek Flow. This enabled us to identify all mutations found within the dataset and finally extract all gene variants that deviated from the reference genome.

### 2.3. Determining Carcinogenicity of the Gene Variants

In both cases, AutoEpiCollect 2.0 organized carcinogenic variants from the most frequent to the least frequent. The first 20 carcinogenic gene variants were selected from each sample. Each gene was determined to be carcinogenic if the Human Protein Atlas labeled the gene as a prognostic marker in any cancer (Table 2). For case 2, the summary of the top carcinogenic genes is provided in Supplementary Materials File S4. These genes were identified in COSMIC as commonly associated with breast cancer mutations. AutoEpiCollect 2.0 filtered our output data for these gene variants.

We found that the gene AHNAK nucleoprotein 2 (AHNAK2) had the highest mutation frequency across all samples in both cases during our analysis. In case 1, we observed 13, 17, 32, 10, and 5 AHNAK2 variants within our CIN1, CIN3, cancer stage IA1, cancer stage IIB, and control samples, respectively. In case 2, the five tumor samples and their associated number of mutated genes and SNPs were as follows: GSM7105215 was observed to have 13 SNPs across 9 genes. GSM7105217 was seen to have 25 SNPs across 11 genes. GSM7105225 was observed to have 12 SNPs across 10 genes. GSM7105247 was observed to have 15 SNPs across 7 genes. Finally, GSM7105249 was observed to have 13 SNPs across 6 genes. These numbers do not include the statistics for the AHNAK2 gene.

### 2.4. Determining Top Epitopes in Protein Expressed by Carcinogenic Gene Variants

We compiled a ranked list of the top 50 epitopes with the greatest potential for each sample, based on binding affinity, allergenicity, immunogenicity, and antigenicity parameters collected by AutoEpiCollect 2.0. In total, there were 4671 MHC class I epitopes and 20,871 MHC class II epitopes identified for case 1 (Table 3). In case 1, three of the top four MHC class I epitopes were observed to be targeted to the heparan sulfate proteoglycan 2 (HSPG2) variants. The remaining top four epitopes were selected for the mechanistic target of rapamycin kinase (MTOR) variant. We also see the mucin 16 (MUC16) variant amongst the top epitopes, comprising 26% of the top 50 epitopes. The Fc gamma binding protein (FCGBP) variant demonstrated high prevalence, with 12% of the top 50 epitopes focused on FCGBP variants. Other recurring variants include WD repeat domain 11 (WDR11), mucin 6 (MUC6), and lipoprotein receptor-related protein 1 (LRP1). The AHNAK2 and titin (TTN) were noteworthy variants that were seen to appear within the top 50 epitopes. Among the top epitopes for MHC class II, 47 out of 50 targeted the mucin 5AC (MUC5AC) variant (Table 4). Two epitopes targeted exophilin 5 (EXPH5) variants, and one epitope targeted HECT and RLD domain-containing E3 ubiquitin protein ligase family member 1 (HERC1).

In case 2, the top 50 MHC class I epitopes were observed to contain a substantial number of AHNAK2 records with BRCA2 as the top epitope seen in our pre-menopausal HER2(−)/ER(+)/PR(+) sample. The epitope with the greatest ability to generate an immune response was identified as the rank 23 epitope for PIK3CA (Table 5). In terms of antigenicity, the rank 36 epitope corresponding to ERBB4 was determined to have the greatest ability to bind T cell receptors (TCRs). Interestingly, the rank 38 epitope corresponding to AHNAK2 had the greatest ability to generate an allergic response. Conversely, rank 47, which also corresponds to AHNAK2, was seen to have the greatest logarithm of binding affinity value. As mentioned previously, case 1 also had AHNAK2 mutations across all samples. However, these variants did not result in any high-potential epitopes within the top 50 epitopes.

The same procedure was repeated to obtain possible epitopes for MHC class II molecules. In sample GSM7105215, the top 8 epitopes for MHC class II were within the gene AHNAK2 (Table 6). This reinforces the concept that the AHNAK2 gene may be involved in tumorigenic processes and may serve as an ideal candidate for the peptide vaccine.

We determined the rank 1 epitope for each of the samples of both MHC class I and II molecules (Table 7). AutoEpiCollect 2.0 determined the epitope with the greatest potential for the five tumor samples, and we observed a repeat in results with three out of the five samples. GSM7105217, GSM7105215 and GSM7105249 showed that the top MHC class I epitope for the BRCA2 variant V2466A epitope QAAVTFK binding to HLA allele HLA-A*68:01. These samples also shared the same MHC class II epitope within gene AHNAK2 variant G5139E epitope LSTEGDSRGCGLEDV on HLA allele HLA-DQA1*03:01/DQB1*03:02. The samples vary on epitope potential which suggest differences in antigenicity, immunogenicity, binding affinity and/or allergenicity.

### 2.5. Predicting Structures and Interactions Between MHC-Epitopes and MHC-Epitopes-T Cell Receptors

**Figure 2:**
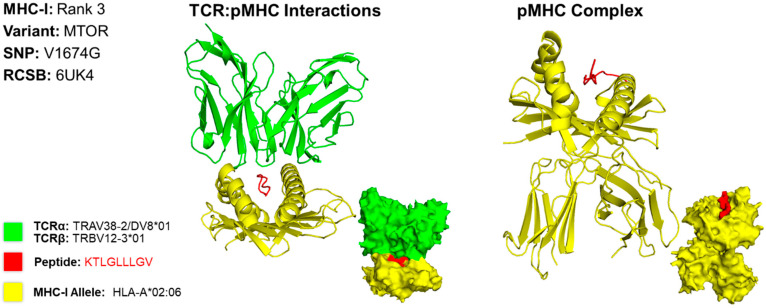
Three-Dimensional Protein Structure for MHC Class I Rank 3 Epitope for CIN3. Richardson diagrams are ribbon models representing protein structures and are determined using X-ray crystallography. This figure illustrates the TCR:pMHC as well as the pMHC interactions with epitope potential represented in red, HLA alleles represented in yellow, and their corresponding TCR represented in green. The smaller image is a surface representation of the same protein. X-ray crystallography structures from UniProt for TCRα/β and MHC-I alleles were leveraged to predict protein interactions. For conformational accuracy, we selected the top third-ranked epitope derived from MTOR due to uncertainty with the TCRα/β and MHC-I alleles that bind to the top first-ranked HSPG epitope. Three-dimensional models for TCR:pMHC generated using TCRmodel and pMHC using HPEPDOCK 2.0. The 3D coordinate PDB file can be found in Supplementary File S9.

**Figure 3:**
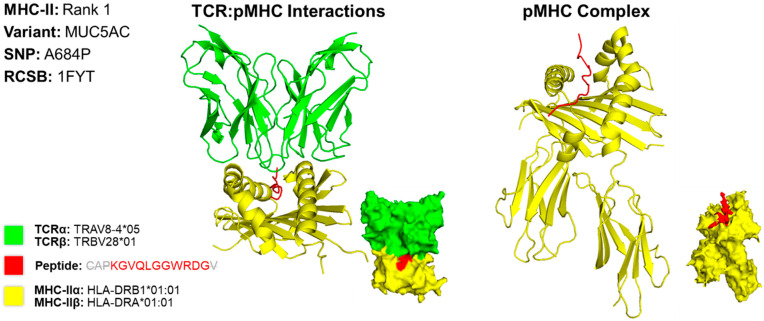
Three-Dimensional Protein Structure for MHC Class II Rank 1 Epitope for CIN3. Richardson diagrams are ribbon models representing protein structures and are determined using X-ray crystallography. This figure illustrates the TCR:pMHC as well as the pMHC interactions with epitope potential represented in red, HLA alleles represented in yellow, and their corresponding TCR represented in green. The smaller image is a surface representation of the same protein. X-ray crystallography structures from UniProt for TCRα/β and MHC-IIα/β alleles were leveraged to predict protein interactions. Three-dimensional models for TCR:pMHC generated using TCRmodel and pMHC using HPEPDOCK 2.0. Note that the 15-amino acid residue antigen was truncated to an 11-amino acid residue antigen by TCRmodel. The 3D coordinate PDB file can be found in Supplementary File S10.

We predicted the structure of the top epitope for MHC classes I and II for both cases. For case 1, we used a PDB file containing the X-ray structure of HLA-A*02:06 [13]. The MHC class I epitope with the greatest potential contained an amino acid residue sequence of KTLGLLLGV. This sequence targets the MTOR variant caused by a missense mutation at the 1674th position, resulting in a substitution of valine for glycine (V1674G) (Figure 2). The rank 3 epitope targeting MTOR was selected for MHC class I due to the variation in structures found in scientific literature on what the HLA allele structures look like for the higher-ranked epitopes targeting HSPG2. For conformational accuracy, we selected the 3rd-ranked epitope due to its concordance with the protein structure across scientific literature. For MHC class II, we used the X-ray structure of HLA-DR1 complexed to an influenza peptide and a human alpha/beta TCR [14]. Our MHC class II epitope with the greatest potential contained an amino acid residue sequence of CAPKGVQLGGWRDGV. This sequence targets the MUC5AC variant caused by a missense mutation at the 684th position, resulting in a substitution of alanine for proline (A684P) (Figure 3).

**Figure 4:**
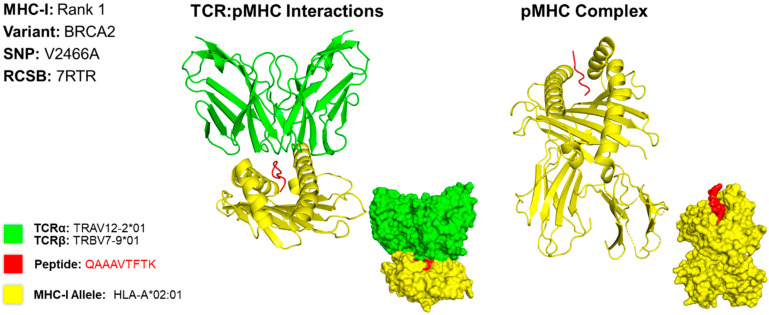
Three-Dimensional Protein Structure for MHC Class I Rank 1 Epitope for GSM7105215. The 3D model of the top MHC class I epitope represents protein structures and is determined using X-ray crystallography. This figure illustrates the TCR:pMHC as well as the pMHC interactions. The smaller image is a surface representation of the same protein. X-ray crystallography structures from UniProt for TCRα/β and MHC class I alleles were leveraged to predict protein interactions. We selected the top ranked epitope derived from the BRCA2 variant V2466A epitope QAAAVTFK binding to HLA allele HLA-A*68:01. Three-dimensional models for TCR:pMHC generated using TCRmodel and pMHC using HPEPDOCK 2.0. The 3D coordinate PDB file can be found in Supplementary File S11.

For case 2, we developed a 3D model for the MHC class I epitope QAAAVTFK, which is located within the BRCA2 gene variant caused by a missense mutation at the 2466th position, resulting in a substitution of valine to alanine (V2466A) (Figure 4). We determined that the PDB file containing the X-ray crystallography for HLA allele HLA-A*68:01 produces the best binding affinity for this epitope. However, a structure for HLA-A*68:01 was not available; thus, HLA-A*02:01 [15]. was used as it is structurally and functionally similar to HLA-A*68:01 [16]. The T cell receptor TRAV12-2*01/TRBV7-9*01 [15]. used to model this interaction is known to interact with the HLA allele used for modeling [17].

**Figure 5:**
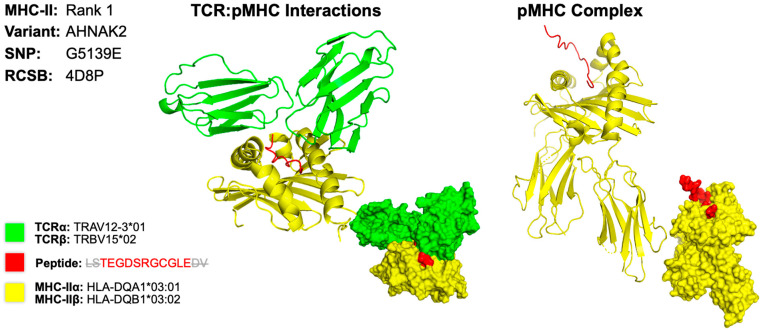
Three-Dimensional Protein Structure for MHC Class II Rank 1 Epitope for GSM7105215. The 3D model of the top MHC class II within representing protein structures is determined using X-ray crystallography. This figure illustrates the TCR:pMHC as well as the pMHC interactions. The smaller image is a surface representation of the same protein. X-ray crystallography structures from UniProt for TCRα/β and MHC class II alleles were leveraged to predict protein interactions. We selected the top ranked epitope derived from the AHNAK2 variant G5139E epitope LSTEGDSRGCGLEDV on HLA allele HLA-DQA1*03:01/DQB1*03:02. Three-dimensional models for TCR:pMHC generated using TCRmodel and pMHC using HPEPDOCK 2.0. Note that the 15-amino acid residue antigen was truncated to an 11-amino acid residue antigen by TCRmodel. The 3D coordinate PDB file can be found in Supplementary File S12.

The 3D model for the MHC class II epitope LSTEGDSRGCGLEDV was identified within the gene AHNAK2, which is caused by a missense mutation at the 5139th position, resulting in a substitution of glycine for glutamate (G5139E) (Figure 5). We determined that the HLA allele HLA-DQA1*03:01/DQB1*03:02 produces the best binding affinity for this epitope. For this 3D model, we encountered some difficulty finding PDB files that contained the X-ray crystallography that matched the HLA and TCR to represent our results. As an alternative, we used the dropdown menu from the TCRmodel [18]. website and selected the mentioned HLA genes which provided the corresponding RNA sequences for the HLA peptides. The T cell receptor TRAV12-3*01/TRBV15*02 was used to demonstrate T cell activation because it closely interacts with HLA alleles mentioned [19]. The sequence used to determine the X-ray crystallography for TCR was identified on the Uniprot [20]. website by typing in the separate α and β subunit gene name. The final 3D model was obtained by entering the respective TCR subunit sequence, selecting the correct MHC gene from the dropdown, and entering the identified peptide antigen.

The 3D models show there is a close relationship between the identified epitope, MHC HLA allele, and TCR molecule. This suggests that the epitopes we have identified in this case can mount an immune response directed against a neoantigen isolated from the original five tumor samples.

## 3. Discussion

In this study, we designed personalized peptide cancer vaccines for CSCC and BC using the innovative data integration capability in AutoEpiCollect 2.0. We successfully identified our top epitopes for MHC class I and MHC class II based on the antigens synthesized by the carcinogenic variants in our cervical carcinoma and BC samples. These antigens form the basis for our peptide cocktail vaccine that can then be used to prime the adaptive immune system and mount highly specific immune responses against all cancer cells expressing these neoantigens.

The ability to identify and prioritize these epitopes was significantly improved by the features of AutoEpiCollect 2.0. The most transformative improvement in AutoEpiCollect 2.0 compared to its predecessor is the introduction of two distinct processes for gene selection. The separate gene selection processes improve the flexibility of epitope identification for personalized vaccine development. As described in Section 2.1, users now have the ability to choose between selecting epitopes with general carcinogenic potential and cancer-specific genes. This update allows for more targeted vaccine formulation strategies, a critical step toward personalized medicine and the development of more effective cancer immunotherapies. In addition to this major improvement, AutoEpiCollect 2.0 also offers several other enhancements over AutoEpiCollect 1.0. The transition to a cloud-based platform hosted on Streamlit is a key change that improves the tool’s accessibility and usability. Unlike the previous version, which required users to manually redownload updates and bug fixes, users now automatically receive updates for AutoEpiCollect 2.0. This transition guarantees that all users operate on the most current and stable version of AutoEpiCollect 2.0.

Another major upgrade is the ability to process larger datasets using Streamlit’s cloud infrastructure. AutoEpiCollect 2.0 can now handle millions of genetic variants from Partek Flow without relying on the user’s local computing resources. This frees the user’s system for other tasks and prevents interruptions from background processing. The introduction of two distinct processes for gene selection further enhances the tool’s flexibility. By integrating a more user-driven approach to epitope selection, AutoEpiCollect 2.0 supports greater specificity in cancer vaccine design. This improvement advances the development of more targeted and effective immunotherapies.

Using the new upgrades for AutoEpiCollect 2.0, we determined the potential for all MHC class I and II epitopes to bind T cell receptors and initiate immune responses. The top-ranked epitope for MHC class I in case 1 was heparan sulfate proteoglycan 2 (HSPG2), a protein that HPV preferentially recognizes and attaches itself to (Table 3). According to Xie et al., high expression of HSPG2 always indicates the disease invasion, metastasis, and angiogenesis of solid tumors [21]. Additionally, the top-ranked MHC class II epitope for the CIN3 sample of case 1, as captured by Table 1, was mucin 5AC (MUC5AC). Interestingly, HPV may be upregulating the top MHC class I infection-permissive HSPG2 variant while downregulating the top MHC class II infection-protective MUC5AC variant.

**Figure 6:**
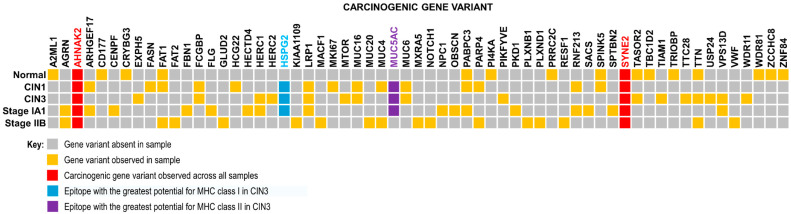
Gene Variant Presence Heatmap. Heatmap showing prevalence of specific gene variants found across samples of cervical squamous cell carcinoma. Analysis of five different tumor stages resulted in 63 common carcinogenic gene variants. Frequently mutated genes include heparan sulfate proteoglycan 2 (or HSPG2), a prognostic marker in urothelial cancer, LDL receptor related protein 1 (or LRP1), a prognostic marker in both urothelial cancer and renal cancer, and mucin 5AC (or MUC5AC), a gene often mutated in pancreatic and gastric cancers. Mutations in neuroblast differentiation-associated protein 2 (or AHNAK2) and spectrin repeat-containing nuclear envelope protein 2 (or SYNE2) genes were seen across all samples, including the morphologically normal tissue sample. AHNAK2 mutations are prognostic markers for pancreatic, urothelial, and lung cancers, while SYNE2 mutations are prognostic markers for renal cancer. Mutations in AHNAK2 and SYNE2 span across all samples. Other high frequency mutations include FAT1, HSPG2, LRP1, MUC5AC, PABPC3, and TTN. Note the presence of gene variants found in the morphologically normal tissue (control) sample as well.

The morphologically normal tissue sample in case 1 exhibited eight missense mutations within the TTN gene, producing the titin protein. The TTN variant was seen to be a highly recurring variant appearing in three out of five samples for case 1 (Figure 6). Other studies have found significant recurrent mutations in TTN to the point that the most frequently mutated gene in cervical cancer has been identified as TTN [22]. Additionally, increased mutational burden within the same gene serves as an indicator for genomic instability and defective DNA repair mechanisms. With its composition of 34,350 amino acids, the titin protein has a higher probability of incurring missense mutations in the scenario of genomic instability [23]. Hence, TTN may be used as a marker for genomic instability as the gene acquires increased tumor mutation burden, a hallmark of many aggressive cancers [23]. This suggests that the several mutations observed in the TTN variant of our morphologically normal control sample also demonstrate genomic instability and inevitably lead to the development of cancer in the future.

HPV has been widely accepted by the scientific community as the etiological agent for cervical cancer. With Gardasil 9 providing prophylactic protection against HPV infection, cases of cervical cancer caused by chronic HPV infection are expected to decline. Therefore, the need for a personalized peptide vaccine targeting cervical cancer may seem redundant and unnecessary. However, there is still the possibility of acquiring high-risk mutations leading to spontaneous cervical cancer development. These spontaneous cases may have been confounded or obscured by HPV-related cervical cancer cases before the introduction of prophylactic HPV vaccines. Pertaining to our study, we found TTN mutations in CIN3 and stage IIB samples, as well as our health control sample. Therefore, an acquired mutation in the TTN gene may still result in a spontaneous case of cervical cancer. Evidence of a TTN variant in the control sample suggests that some individuals may currently have high-risk mutations, but are otherwise healthy and do not present diagnostically with cancer. Therefore, we believe that there is still a niche requirement for the personalized peptide vaccine in the spontaneously acquired cancer population, as well as the population of healthy individuals who currently have high-risk mutations. More information on the need for personalized peptide cancer vaccines for cervical cancer can be found in Supplementary File S13.

**Figure 7:**
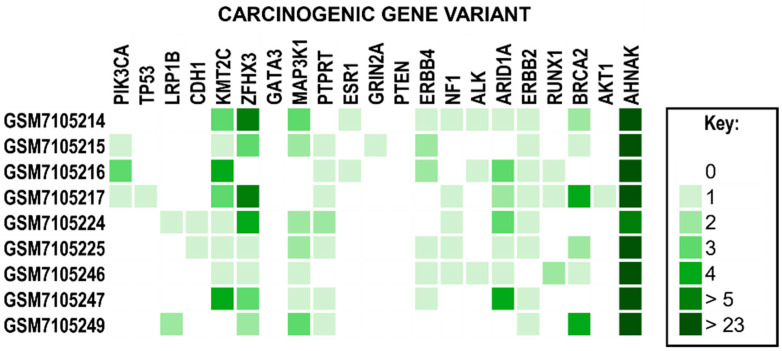
A Heatmap of Common Breast Cancer Gene Variants. Genes have been identified as gene variants associated with breast cancer. The legend on the right describes the color associated with the number of unique variants. Note that all samples observed the highest frequency of mutations within the AHNAK2 gene variant.

Case 2 resulted in three out of the five analyzed tumor samples with the genetic profiles of premenopausal HER2(−)/ER(+)/PR(+), post-menopausal HER2(−)/ER(+)/PR(+) and pre-menopausal TNBC having the top ranked epitope for MHC class I as BRCA2 (Figure 7). BRCA2 is a tumor suppressor gene involved in DNA replication and, specifically, homologous recombination DNA repair. BRCA2 protein promotes and stabilizes Radiation sensitive 51 (RAD51) onto single-strand DNA (ssDNA) at the damaged site [24]. Cells expressing a mutation in this protein will facilitate genomic instability and allow for replication of mutated DNA in subsequent generations. As the name of the gene suggests, this gene has been known to serve as a prognostic marker for breast cancer. The current standard of care screens for women who may inherit this mutant gene [25]. Additionally, the sample GSM7105225 had the top-ranked epitope for MHC class I of erythroblastic oncogene B (ERBB2), which is a receptor tyrosine-protein kinase now known as HER2. This gene is a protooncogene that aids particularly in the proliferation of breast cancer by upregulating expression on the surface of tumor cells [26]. HER2 is also a known prognostic marker for the development of breast cancer. BRCA2 and HER2, both being broadly known to be associated with breast cancer, serve as a point of confirmation of the efficacy of AutoEpiCollect 2.0. The MHC class II top-ranked epitope for all five samples showed AHNAK2, which is the novel protein that we identified as the most frequently mutated throughout all samples, as well as in case 1. This protein plays a role in many different functions, particularly with the fibroblast growth factor 1 (FGF1) gene function in cell growth and tumor invasion [27].

AHNAK2 is a gene that is still being understood, but has been shown to be closely associated with cancer biology. AHNAK2 shows promise as a target likely for its role in cell structure and migration, as well as cell signaling pathways related to cell growth, differentiation, and survival [28]. A study by Xu et al. suggests that AHNAK2 not only serves as a biomarker for adenocarcinoma but may also serve as a potential therapeutic target [28]. In addition, AHNAK2 is highly expressed in glandular tissues, which include both cervical cancer and breast cancer [28]. The prevalent nature of AHNAK2 in glandular tissue provides the potential opportunity to use AHNAK2 neoantigens as a target for multisystem adenocarcinomas. Notably, the knockout of the AHNAK2 gene showed a diminished tumor progression, further confirming AHNAK2’s role in tumorigenesis. It is of significance that all tumor samples in case 2 not only included this gene as the highest frequency of mutation but also shared many of the exact same mutations. This may suggest AHNAK2 plays a larger role in tumorigenesis. Given its critical role in proliferation, migration, DNA replication, and apoptosis, we have chosen to include this gene in our analysis as an auxiliary target for a peptide vaccine. It was the highest frequency of mutations in case 2, and the observed mutational prevalence across different cancer types. Our findings suggest that the addition of epitopes derived from AHNAK2 variants will improve personalized peptide vaccine efficacy and serve as the starting line for the development of a pan-cancer vaccine.

Furthermore, tumor microenvironments and tumor heterogeneity may cause the downregulation or loss of HLA class I antigens in cancer cells, which allows evasion from T cell surveillance. In a study by Giatromanolaki et al., a third of BCs suffer from the coordinated loss of HLA class I, β2-microglobulin, and TAP1 expression. These are all components necessary for antigen presentation [29]. Consequently, peptide cancer vaccines must be administered early when tumor heterogeneity is minimal and microenvironmental conditions are less aggressive.

Tumor heterogeneity also adds complexity by rendering ICIs effective in some tumor cells more than others due to changes in protein expression. Patients may see initial cancer regression, but tumor heterogeneity leads to the selection of resistant tumor cells and the inevitable progression of cancer. Personalized peptide vaccines provide a solution to this problem by leveraging a peptide cocktail of antigens that targets multiple mutant proteins on cancer cells simultaneously. Using AutoEpiCollect 2.0, we produced a robust list of ranked antigens expressed on tumor cells, serving as multiple potential targets. For example, in our study, the top antigens were BRCA2, ERBB4 and AHNAK2 in sample GSM7105215. This confirms these antigens as not only cancer-associated but also effective candidates as therapeutic targets. This multi-antigen approach would include several of our top antigens to produce a peptide cocktail of antigens. This competes with the ability of these resistant tumors to hide immune targets. This overwhelms their ability to adaptively respond by downregulation of specific proteins. As a form of active immunotherapy, peptide vaccines leverage the body’s immune system to train and invoke a focused de novo immune response against selected tumor-specific antigens (TSAs). As a result, the immune system can eliminate targeted pathogens while developing long-term humoral immunity [30]. maintained by memory B cells. Additionally, the targeted therapeutic approach of peptide vaccines spares healthy cells, unlike traditional chemotherapy, which indiscriminately destroys both healthy and tumorigenic cells.

When considering vaccine efficacy, we must consider when mutations occur relative to the abnormal cell replication process. Whole genome doubling (WGD) is associated with the development of cancer as cells undergo polyploidization. Cells may enter a tetraploid state rather than the normal diploid state resulting in chromosomal instability. This instability enables genetic plasticity which leads to the evolution of cancer [31]. Furthermore, pre-WGD mutations result in the multiplication of mutations across the multiple copies of chromosomes and permitting these mutations to be more resilient. In contrast, post-WGD mutations are present on a single copy of the duplicated genome [32]. Therefore, pre-WGD mutations serve as more reliable targets for vaccines due to being found in all cancer cells. Future cancer vaccines should focus on neoantigens formed from pre-WGD mutations to minimize the risk of immune escape by cancer cells. If our peptide vaccine targets pre-WGD events, we may be able to slow down or stop the progression of genetic plasticity, thereby inhibiting cancer cell evolution and increasing patient prognosis. Mutations that occur in certain genes involved in genomic stability, such as BRCA2 and ERBB2 found in GSM7105215 and GSM7105225, respectively, may predispose individuals from initiating an WGD event. Therefore, inclusion of these antigens in the peptide cocktail vaccine would prevent carcinogenic progression due to cell polyploidization. We can expect an amplified response to the personalized vaccine as there is an increase in the overall neoantigen load.

**Figure 8:**
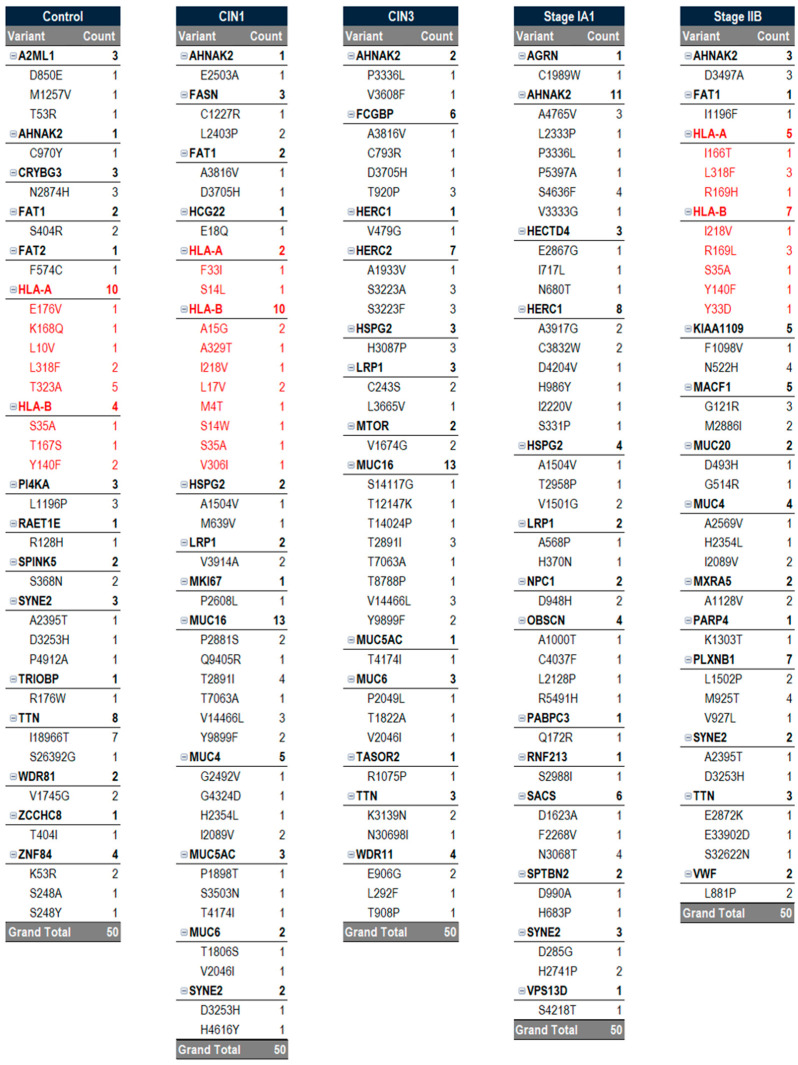
Vaccine Components Per Sample. Each sample used in this case study is vertically represented and captures all 50 epitopes that were selected based on their potential. All samples identified 50 epitopes, but each sample may have varying numbers of gene variants as certain variants had more missense mutations within them than others. Personalized peptide vaccines consist of a cocktail of peptide variants generated by cancer-prognostic gene mutations. Therefore, a personalized peptide vaccine would include antigens for each of the missense mutations listed in the vertical sample. Potentials were determined using binding affinity, immunogenicity, allergenicity, and antigenicity parameters. Note that HLA-A and HLA-B (indicated in red) were removed from the control, CIN1, and cancer stage IIB samples, as inclusion of these variants may result in a suboptimal immune response or the development of autoimmunity.

Personalized peptide vaccines are designed with the individual patient in mind and customized to the vaccine based on the specific missense mutations observed within their carcinogenic variants. However, the identification of carcinogenic gene variants and the development of personalized peptide vaccines are dependent on tumor biopsy and RNA sequencing becoming a part of the normal protocol. If we consider our CIN3 sample from case 1, the vertical columns represent personalized peptide vaccines for any given patient (Figure 8). The peptide cocktail would have to contain 50 distinct epitopes derived from missense mutations across 13 carcinogenic gene variants. This data should be deidentified and aggregated to enable deeper studies on mutational patterns observed across the population. In case 2, we observed all 5 tumor samples and non-tumor samples had mutations in many of the same genes such as AHNAK2, ZFHX3, KMT2C and MAP3K1 (Figure 7). Implementing a protocol to sequence all breast tumor biopsies will provide more information on what mutational trends are prevalent amongst this population. Collection of tumor RNAseq data leads to the formation of big data, which can be processed, integrated, and analyzed by machine learning algorithms for deeper trends and insights than would otherwise be possible. Using this information, we can curate a peptide vaccine targeting these prevalent mutations for all breast cancer patients. RNAseq data collection and aggregation may ultimately lead to a concept of a generalized population peptide vaccine as more tumor samples are sequenced.

While chemotherapy is effective at eliminating proliferating tumor cells, recurrence remains a significant clinical challenge. One explanation for this is tumor dormancy, a state in which cancer cells remain in the body but persist as clinically undetectable and non-proliferating for an extended period of time. Therefore, cytotoxic chemotherapy efficacy is reliant on regimented drug administration as well as active tumor cell proliferation. In cases where breast cancer progresses to requiring systemic therapy, chemotherapy remains the standard of care. However, instances of recurrence or resistance to chemotherapy remains a challenge, reinforcing the need for adjuvant strategies. In our sample GSM7105215, using a personalized peptide vaccine targeting cells that expresses mutant variants of BRCA2, AHNAK2, ERBB2, or other neoantigens determined using AutoEpiCollect 2.0 could be used following chemotherapy or radiation treatments to target residual tumorigenic cells. Hence, the inclusion of personalized peptide vaccines in cancer treatment regimens could improve the therapeutic efficacy of standard cancer treatments by preventing cancer recurrence via targeted destruction of non-proliferative tumor cells at risk for acquiring a tumor suppressor gene mutation. We propose that augmenting chemotherapy with the personalized peptide vaccine could allow for a two-pronged approach towards targeting both prolific and non-prolific tumor cells, further enhancing therapeutic efficacy. Through its ability to identify the highest potential epitopes, AutoEpiCollect 2.0 allows users to select for only the most potent epitopes capable of generating an effective immunoresponse and thereby improving patient outcomes.

## 4. Materials and Methods

**Figure 9:**
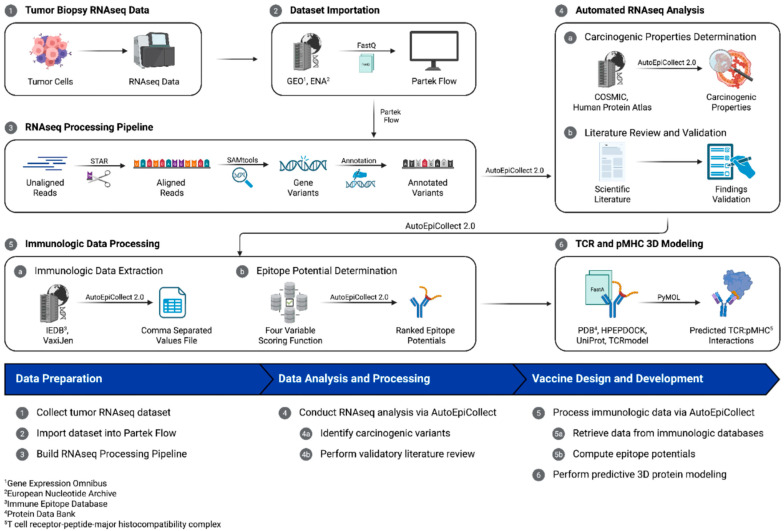
The personalized vaccine design workflow. This study collected tumor biopsy RNAseq data, which was processed within a bioinformatics tool for variant identification and analysis. AutoEpiCollect 2.0, a tool designed to integrate with the bioinformatics tool, automated the data analysis process and filtered output data for expressed genes and missense mutations. AutoEpiCollect 2.0 also automatically collected epigenomic and immunologic data used in determining MHC class I and II epitope potentials.

To demonstrate the flexibility of the personalized vaccine design process, a generalized six-step workflow was implemented for both case 1 and case 2. While the overall process was the same for both cases, each case had distinct features within its respective workflow (Figure 9).

### 4.1. Collecting and Processing Samples of Patient Tumor Data

Patient tumor data containing tissue samples of CSCC were identified and collected from the Gene Expression Omnibus (GEO). We selected datasets GSE223804 [GEO Accession Viewer] and GSE227679 [GEO Accession Viewer] for case 1 and case 2, respectively, because they include samples from different stages or subtypes of CSCC and breast cancer. GSE223804 was a study that explored the molecular differences between high-grade CIN and early-stage invasive CSCC. In this study, RNA sequencing of HPV-positive CIN lesions, CSCC, and normal cervical tissue showed apparent differences in immune responses. GSE227679 refers to the SAGES study, which utilizes structural analysis and bioinformatics to identify genes and proteins associated with breast cancer. Both studies used bulk gene expression profiles for their analyses.

The dataset within GSE223804 comprised 16 surgically obtained tissue samples, encompassing all grades of CSCC. We selected five samples for case 1 from the tumor dataset: CIN1, CIN3, stage IA1, stage IIB, and a morphologically normal control tissue sample. Although the samples used were limited, samples were intentionally selected to be heterogeneous to facilitate the identification of high-risk gene variants present across all samples. Moreover, the use of a limited heterogeneous sample set was sufficient in demonstrating AutoEpiCollect 2.0’s automated genetic analysis capabilities as a proof of concept.

Similarly, we collected breast tumor tissue samples from GSE227679. We chose this dataset because it included a wide range of genetic profiles, menopausal statuses, and treatment records. These were factors relevant to our analysis. Tumor types included HER2(+)/ER(−)/PR(−), HER2(−)/ER(+)/PR(+), and HER2(−)/ER(−)/ER(−). Menopausal status was also considered, as hormone levels could affect immune response and peptide effectiveness. The dataset contained 44 samples total, with 22 from tumor tissue. Of these 22, 10 had no genetic profile listed, two were TNBC, one was HER2(+)/ER(−)/ER(−), and nine were HER2(−)/ER(+)/PR(+).

To analyze the RNAseq data from our samples, we used the NGS capabilities of Partek Flow (San Diego, CA, USA) to build a pipeline that processes our data and identifies the gene variants found within our selected samples. The sample data from GEO were in FASTQ format, a text-based file that contains transcript sequences along with quality scores for each nucleotide. After patient data was imported, the reads were aligned using the Spliced Transcripts Alignment to a Reference (STAR) aligner. The STAR aligner capability is integrated into Partek Flow and functions to align RNAseq data to a common reference genome.

Typically, data is trimmed to remove short sequences attached to RNAseq data in preparation for NGS. Also known as RNA adaptors, these short sequences are oligonucleotides included in RNAseq data to ligate the ends of RNA fragments [33]. Sequencing without trimming RNA sequences may result in the inclusion of these oligonucleotides during RNA sequencing, ultimately leading to a loss of data integrity. However, trimming the data in Partek Flow for our samples resulted in no change in sequence length. Therefore, we determined that the data had already been trimmed and proceeded to align the reads.

Next, we added SAMtools to our pipeline, a bioinformatics software toolkit that is integrated into Partek Flow. SAMtools manages aligned reads by sorting, analyzing, and filtering the data in sequence alignment/map (SAM) format. In addition, SAMtools allows users to identify variations found within genomic data by comparing reads to a reference genome containing normal RNA data. Filtering via SAMtools also removed low-quality reads within our data to ensure that the identified gene variants were accurate and meaningful. In simple terms, SAMtools enabled the pinpointing of the exact locations of variants within our sequence data.

After SAMtools identified the gene variants, the Annotate Variants capability was added to the pipeline to provide more context on the variants identified by SAMtools. Specifically, the Annotate Variants function classifies gene variants by their location (e.g., intron or exon) and the type of change that caused them (e.g., SNPs, insertions, deletions, missense, or nonsense mutations). We then used RefSeq Transcripts 96 to provide standardized and annotated human reference sequences, which include data on genes, transcripts, and proteins. RefSeq Transcripts 96 is a comprehensive database of reference sequences that is maintained by the National Center for Biotechnology Information (NCBI). This provided the basis for our filtering criteria used to analyze the data output by Partek Flow. The final Partek Flow output files for cases 1 and 2 are included in Supplementary Materials Files S1 and S2, respectively.

### 4.2. New Updates in the Development of AutoEpiCollect 2.0

Our previous version of AutoEpiCollect was available as a free, locally downloadable graphical user interface (GUI) software [11]. However, any bug fixes or updates required users to manually redownload the software, making it difficult to stay up to date with the latest improvements. To improve accessibility, usability, and overall appearance, we developed a new web-based version using Streamlit (San Francisco, CA, USA), an open-source Python 3.10 framework. This website is hosted on Streamlit’s cloud, ensuring that all users automatically receive updates as soon as they are deployed. The majority of the back-end functionality for AutoEpiCollect 2.0 is the same as 1.0; however, there are some differences, particularly in the beginning of the workflow to process the raw data from Partek Flow. Before epitope selection, Partek Flow outputs a .csv file listing gene variants with multiple attributes, including gene ID, gene section, and amino acid changes. The updated AutoEpiCollect 2.0 first filters for gene variants located in exons, the protein-coding regions of genes, and then retains only those with missense mutations, excluding other types such as nonsense mutations. Although nonsense mutations can contribute to cancer, we exclude them because they result in premature stop codons, which could trigger unintended autoimmune responses when used in vaccine design. Once filtered, AutoEpiCollect 2.0 processes the Partek Flow output by ranking gene variants according to their frequency, from highest to lowest. The software retains key data such as gene ID, exon number, and missense mutations in standard notation (e.g., A313S). These variants are then sorted by their frequency and categorized into two distinct workflows for epitope identification.

After finishing the Partek Flow data processing, AutoEpiCollect 2.0 now follows two distinct processes that further reduce the number of prospective target genes. These two processes are chosen by the user and labeled as modules 1 and 2, corresponding to cases 1 and 2 defined in this study. Module 1 draws on immunologic data from the Human Protein Atlas to confirm variants and rank those most correlated with carcinogenic mutations in each sample. We chose to keep the top 50 genes with the largest number of carcinogenic variants. Module 2 cross-references variants with the COSMIC database. This allows for the identification of variants specifically associated with particular cancer subtypes. AutoEpiCollect 2.0 web-scrapes the top 20 most frequently mutated genes from COSMIC for a given cancer subtype and retains only those variants that overlap with the Partek Flow list. After identifying the target genes and mutations, AutoEpiCollect 2.0 proceeds to the next stage: the epitope selection process.

### 4.3. AutoEpiCollect Epitope Selection Process

**Figure 10:**
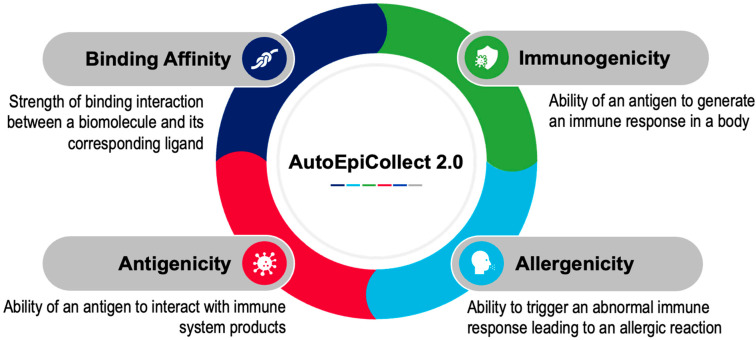
Parameters Used for Determining Epitope Potential. AutoEpiCollect 2.0 determined the potential for MHC class I and MHC class II epitopes within each sample by collecting data in four parameters: binding affinity, immunogenicity, allergenicity, and antigenicity. Antigenicity values are currently not automatically web-scraped due to reCAPTCHA programs. Data within each parameter is normalized to ensure that all parameters contribute to epitope potential proportionately. The top 50 epitopes with the greatest potential were selected for both MHC class I and II.

The epitope selection process in AutoEpiCollect 2.0 remains essentially the same as in the previous iteration, with one small change in the workflow that affects the user. This modification is discussed at the end of this paragraph. After obtaining a list of peptides predicted to be epitopes from IEDB, multiple epitope characteristics are collected from various databases. Four characteristics in particular—binding affinity, immunogenicity, antigenicity, and allergenicity—serve as input features for a machine learning-based ranking model that scores each epitope. These factors were selected because they are consistently associated with epitope immunogenicity in prior literature and are accessible through widely used immunoinformatics tools (Figure 10). Binding affinity values are obtained from NetMHCpan-4.1 and NetMHCIIpan-4.1, which were selected for their high performance in community benchmarks. Immunogenicity scores are collected from IEDB’s immunogenicity prediction modules, antigenicity values are sourced from VaxiJen (Headington, Oxford UK), and allergenicity predictions are obtained from AlgPred (Class I) and NetAllergen (Class II). These tools were chosen for their extensive experimental validation and broad adoption in computational vaccinology. The only change to the epitope selection process in AutoEpiCollect 2.0 is that antigenicity values are no longer automatically web scraped from VaxiJen, as VaxiJen now employs a reCAPTCHA system that blocks automated data retrieval. Although this does not affect the machine learning workflow, users must now obtain antigenicity results manually using the preliminary output generated by AutoEpiCollect 2.0. More information on how this step is carried out is provided in Section 2.1, which discusses the new website’s usability.

Before model training and prediction, the four main epitope characteristics are normalized to make their scales comparable. Immunogenicity, antigenicity, and allergenicity values undergo z-scoring followed by min–max scaling to a 0–1 range. Binding affinity values, which span multiple orders of magnitude, are log-transformed before normalization to reduce the influence of extreme values. These normalized features constitute the input data for each epitope processed by the machine learning model. The machine learning framework used for ranking is a probabilistic logistic regression model. This model was trained on binary T cell assay outcomes from NEPdb for MHC Class I epitopes and from IEDB for MHC Class II epitopes. Logistic regression was selected because its probability output aligns with our goal of producing an interpretable immunogenicity likelihood and because it outperformed the linear model previously tested. In cross-validation experiments performed during the development of AutoEpiCollect 1.0, the logistic model demonstrated higher accuracy than the linear model and produced more stable and biologically consistent rankings. Using the learned regression weights, AutoEpiCollect 2.0 computes a probability for each epitope that reflects how likely it is to serve as an effective immunogenic candidate. Only the top-ranking epitopes proceed to the downstream filtering step, which applies cutoff values to characteristics such as instability, half-life, and toxicity. A complete description of the epitope selection process—including how each epitope characteristic was obtained, the design and final weights of the logistic regression model, and the specific exclusion criteria used during manual filtration—is provided in the AutoEpiCollect 1.0 manuscript [11].

### 4.4. Using AutoEpiCollect 2.0 for Personalized Vaccine Design

For the personalized vaccine design performed in this study, we began with Partek Flow output files containing gene variants for each patient sample. AutoEpiCollect 2.0 first filtered these variants to include only missense mutations in coding regions, as detailed above. After filtering, AutoEpiCollect 2.0 ranked the remaining missense variants by their frequency and retained essential information including gene ID, exon number, and the specific amino acid substitutions. Our study used the two gene filtering methods implemented within AutoEpiCollect 2.0, modules 1 and 2. Modules 1 and 2 were used for cases 1 and 2, respectively. After selecting the relevant genes and mutations for each case, AutoEpiCollect 2.0 automatically generated all possible Class I and Class II peptides surrounding the mutations and collected the four parameters used to compute epitope potentials: binding affinity, immunogenicity, allergenicity, and antigenicity. These parameters were normalized within the software so that each characteristic contributed proportionately to the potential calculation. Using these normalized characteristics, AutoEpiCollect 2.0’s machine learning model scored and ranked each epitope. The top 50 Class I and top 50 Class II epitopes were retained for downstream evaluation. For case 1, epitopes were filtered further to exclude those derived from HLA-A and HLA-B proteins. This ensures that the final list of epitopes is tailored to the specific samples under study. The CIN3 sample was used as proof of concept for the analysis. For case 2, each tumor sample was analyzed to identify SNPs within tumorigenic genes, ranking them by the number of commonly mutated genes and total SNPs per gene. This approach also accounted for genetic diversity and excluded treated samples to avoid confounding factors. We selected 5 sample profiles for the study with differing menopausal status and therapeutic interventions (Table 8). Finally, AutoEpiCollect 2.0 generated a detailed output containing an aggregated spreadsheet of all filtered and ranked epitopes, along with population coverage results.

### 4.5. Three-Dimensional Modeling of Top Epitopes

We performed peptide-protein three-dimensional (3D) modeling and docking using HPEPDOCK 2.0 (Zhongshan District, Wuhan, China) [34], a web server that uses molecular docking algorithms to determine biomolecular interactions. This allows for the prediction of the structure and docking of the tumor protein to both MHC classes I and II (pMHC-I/II). HPEPDOCK 2.0 uses receptor input by sequences in fasta format or by Protein Data Bank (PDB) files. We also used TCRmodel (Rockville, MD, USA) [35], a web server that predicts and models TCR structures and docking, to visualize the TCR and pMHC interactions and anticipate their structures based on TCR alpha-chain, beta-chain, peptide, and MHC class I and II sequences. Similarly to HPEPDOCK 2.0, TCRmodel also allows the uploading of FASTA files that contain sequences. In essence, 3D modeling was performed to demonstrate the close relationship between the MHC class I and II molecules, TCR, and their corresponding epitopes.

## 5. Conclusions

The purpose of this study was to investigate case studies on the development of a personalized peptide vaccine for two distinct types of cancer using the innovative data integration capability of AutoEpiCollect 2.0. AutoEpiCollect 2.0 improves upon its predecessor through two new gene selection modules that let users import data directly from Partek Flow and choose between different types of gene analysis. AutoEpiCollect 2.0 automates the analysis of genetic variants from a specified cancer sample and generates a list of ranked epitopes. Epitopes with the highest potential are then curated for inclusion into the design of the personalized peptide cancer vaccine. The transition to a cloud-based Streamlit platform additionally removes the need for manual installation or updates. It also enables the software to handle large genomic datasets without straining the user’s computing power. Together, these updates make AutoEpiCollect 2.0 more flexible, user-friendly, and scalable for larger and more diverse datasets.

Personalized peptide vaccines are also still a relatively new therapy option. While AutoEpiCollect 1.0 shortened immunogenic data collection and determination of optimal epitopes, the development of custom peptides requires time. When pitted against more aggressive forms of cancer, peptide cancer vaccines become a race against survival. Currently, peptide vaccine development still takes longer than the simple administration of traditional cancer therapies such as chemotherapy, immunotherapeutic agents, or surgical excision. AutoEpiCollect 2.0 shortens vaccine development timelines even further by automating the variant analysis and carcinogenic gene identification process.

Efficacy of AutoEpiCollect 2.0 was assessed through the development of personalized peptide vaccines for CSCC and BC which both have their own unique and distinct pathogenesis, progression, and treatments. The CSCC studied in case 1 was selected for its indolent progression, making it a suitable option for exploring the fundamental capabilities of protein engineering in peptide vaccine development. Case 1 leveraged module 1 in AutoEpiCollect 2.0 which identified and prioritized gene variants known to cause cancer. We found HSPG2 and MUC5AC variants for MHC class I and II, respectively. As the etiologic agent for CSCC, HPV may upregulate the top MHC class I infection-permissive HSPG2 variant while downregulating the top MHC class II infection-protective MUC5AC variant. Additionally, we were able to confirm that the top epitopes we identified were indeed high fidelity due to the inclusion of a TTN variant, a gene that has been identified in scientific literature as the most frequently mutated gene in cervical cancer.

In comparison, the breast cancer studied in case 2 was selected for its predictability and the potential to impact one of the more heavily studied cancers within the scientific community. Case 2 leveraged module 2 in AutoEpiCollect 2.0 which identified variants specifically associated with particular cancer subtypes. We observed BRCA2 and AHNAK2 variants for MHC class I and II, respectively. The BRCA2 variant is a known carcinogenic tumor suppressor gene biomarker that normally involves DNA replication machinery. AHNAK2 is the novel protein that we identified as the most frequently mutated throughout all samples, as well as in case 1. In all CSCC and BC samples, MHC class I and MHC class II top epitopes were successfully identified. Furthermore, we identified the highest potential epitopes synthesized by carcinogenic gene variants using our personalized cancer vaccine design.

Our two complementary approaches, using broad prognostic markers in case 1 and cancer-subtype-specific markers in case 2, demonstrated the flexibility and novelty of AutoEpiCollect 2.0 as an automated genetic analysis and epitope-ranking framework. Although the tool successfully identified high-potential epitopes in both cancers, a key limitation of this study is that patient-specific HLA allele expression data were not available. All binding predictions relied on IEDB reference alleles, which do not represent the full diversity of HLA types across global populations. Incorporating patient HLA specificity and expanding allele representation will be essential for increasing vaccine personalization and improving predictive accuracy in future studies. It is also important to note that this work focuses on validating AutoEpiCollect 2.0 as a functional methodology rather than validating the biological efficacy of individual epitopes. The 3D structural models included here serve as illustrations of potential interactions but do not confirm dynamic stability. Molecular dynamics simulations remain the appropriate next step for evaluating epitope viability, and these will be incorporated into future outcome-based studies once a refined shortlist of top candidates is selected. This study also highlights broader methodological considerations. AutoEpiCollect 2.0 currently relies on Partek Flow for preprocessing and variant calling, which may limit accessibility for users without bioinformatics training or access to proprietary tools. Although this reliance ensured high-quality upstream processing in the present work, we recognize the importance of broadening accessibility. In future iterations, we plan to incorporate a fully open-source preprocessing pipeline, using tools such as STAR or HISAT2 for alignment, GATK or Mutect2 for variant calling, and VEP or SnpEff for annotation, to eliminate the need for Partek Flow and allow users to process raw FASTQ files directly within an open framework.

Our case studies focus on the conceptual understanding of how peptide vaccines may function as therapeutics. Concepts such as tumor dormancy may impact peptide vaccine efficacy, as dormant tumor cells containing carcinogenic gene variants remain latent and prevent the active synthesis of altered proteins. This suggests that there may be minimal maintenance of humoral immunity by memory B cells if and when these dormant tumor cells transition to a prolific state. Further in vivo studies are required to explore the potential of peptide vaccines as an alternative option for cancer therapy. Next steps would be to collaborate with translational medicine to put our methodology into medical practice.

Moreover, the peptides selected for incorporation into the peptide vaccines in both cases were not identified as being either pre-WGD or post-WGD. As discussed earlier in Section 5, pre-WGD mutations are more reliable targets for vaccine therapies due to their stability. Pre-WGD mutations are clonal and found in all cancer cells, making them less prone to deletion. Meanwhile, post-WGD mutations exhibit instability and are more easily lost, which leads to therapy resistance. Therefore, pre-WGD mutations would serve as more reliable neoantigen targets in personalized cancer vaccine design. According to Bakir et al., it is possible to identify mutations that are pre-WGD or post-WGD using various analysis techniques including computational timing models such as COrrecting Noise In PHylogenetic Evaluation and Reconstruction (CONIPHER), PyClone, and CNApy [32]. CONIPHER utilizes phylogenetic modeling to infer cancer evolutionary history while PyClone and CNApy uses variant allele frequency analysis and copy number analysis, respectively. With this in mind, expansion of capabilities in AutoEpiCollect 2.0 to include filtration for pre-WGD mutations would allow for the development and design of more persistent and effective neoantigen targets.

Finally, understanding the role of drug delivery systems in enhancing delivery and immunogenicity are critical for improving outcomes associated with cancer peptide vaccines. Advanced drug delivery models allow for innovative strategies to overcome intrinsic limitations of peptide vaccines, such as low immunogenicity, in vivo peptide instability, and rapid elimination and metabolism [36]. One advanced system to note is the use of nanoparticle carriers which encapsulate peptides to protect them from degradation and facilitate targeted delivery to APCs found in lymphoid tissues [37]. Albumin-binding amphiphiles is another modern system used to conjugate peptides to promote lymphatic drainage and accumulation in lymphatic tissues, where immunogenicity occurs. This drug delivery system enhances both protein integrity and serum half-life to prolong antigen presentation and ultimately amplifies T cell priming [38]. Future studies should consider advanced drug delivery models to optimize peptide vaccine efficacy and boost immunogenicity.

## Figures and Tables

**Table 1:** The Number of Reads Pre- and Post-Filtration in Case 2. The table illustrates all samples analyzed and their associated number of reads through filtration steps. The first column, titled “Total Reads,” shows the total final variants obtained from Partek Flow for each of the nine samples analyzed. The second column, titled “Variants Pre-Filtration,” shows the total reads before filtration, which includes all possible variants. The third column, titled “Variants Post-Filtration,” shows the total number of variants after filtration, which only includes single-point mutations within exons causing a missense mutation. The PartekFlow RNAseq output files for GSM7105215, GSM7105216, GSM7105217, GSM7105225, GSM7105247, and GSM7105249 samples used in case 2 can be found in Supplementary Materials Files S19, S20, S21, S22, S23, and S24, respectively.

Sample	Total Reads	Variants Pre-Filtration	Variants Post-Filtration
GSM7105215	56,879,018	993,476	7519
GSM7105216	55,964,917	289,777	6576
GSM7105217	62,017,030	750,867	8228
GSM7105225	55,427,028	496,464	6672
GSM7105247	70,887,673	282,945	7511
GSM7105249	72,776,005	766,817	7294

**Table 2:** Carcinogenic Gene Variants for Case 1. The 25 genes contained in this table generated the top 25 highest potential epitopes. Gene variants were selected based on either their ability to serve as a prognostic marker for a specific type of cancer or its cancer specificity. The full list of carcinogenic gene variants was retrieved from the Human Protein Atlas and can be found in Supplementary Materials File S3.

#	Variant	Description	Prognostic Summary	Cancer Specificity
1	A2ML1	Alpha-2-macroglobulin like 1	Prognostic marker in lung adenocarcinoma, pancreatic adenocarcinoma	Cancer enhanced (head and neck squamous cell carcinoma)
2	AGRN	Agrin	Prognostic marker in liver cancer (unfavorable)	Low cancer specificity
3	AHNAK2	AHNAK nucleoprotein 2	Prognostic marker in pancreatic cancer (unfavorable), urothelial cancer (unfavorable) and lung cancer (unfavorable)	Cancer enhanced (head and neck cancer)
4	ARHGEF17	Rho guanine nucleotide exchange factor 17	Prognostic marker in colorectal cancer (unfavorable)	Low cancer specificity
5	CRYBG3	Crystallin beta-gamma domain containing 3	Prognostic marker in renal cancer (favorable)	Low cancer specificity
6	EXPH5	Exophilin 5	Prognostic marker in endometrial cancer (favorable), head and neck cancer (favorable), renal cancer (favorable) and lung cancer (favorable)	Low cancer specificity
7	FASN	Fatty acid synthase	Prognostic marker in cervical cancer (unfavorable) and renal cancer (unfavorable)	Low cancer specificity
8	FAT1	FAT atypical cadherin 1	Prognostic marker in lung cancer (unfavorable)	Low cancer specificity
9	FAT2	FAT atypical cadherin 2	Gene product is not prognostic	Group enriched (cervical cancer, head and neck cancer, lung cancer, urothelial cancer)
10	FCGBP	Fc gamma-binding protein	Prognostic marker in ovarian cancer (unfavorable), head and neck cancer (favorable), liver cancer (unfavorable) and thyroid cancer (unfavorable)	Cancer enhanced (colorectal cancer)
11	FLG	Filaggrin	Gene product is not prognostic	Cancer enriched (melanoma)
12	HCG22	HLA complex group 22 (gene/pseudogene)	Gene product is not prognostic	Group enriched (glioma, thyroid cancer)
13	HECTD4	HECT domain E3 ubiquitin protein ligase 4	Prognostic marker in head and neck cancer (favorable)	Low cancer specificity
14	HERC1	HECT and RLD domain containing E3 ubiquitin protein ligase family member 1	Prognostic marker in renal cancer (favorable), head and neck cancer (favorable) and pancreatic cancer (favorable)	Low cancer specificity
15	HERC2	HECT and RLD domain containing E3 ubiquitin protein ligase family member 2	Prognostic marker in renal cancer (favorable)	Low cancer specificity
16	HSPG2	Heparan sulfate proteoglycan 2	Prognostic marker in urothelial cancer (unfavorable)	Low cancer specificity
17	KIAA1109	Bridge-like lipid transfer protein family member 1	Prognostic marker in renal cancer (favorable)	Low cancer specificity
18	LRP1	LDL receptor related protein 1	Prognostic marker in urothelial cancer (unfavorable) and renal cancer (unfavorable)	Low cancer specificity
19	MACF1	Microtubule actin crosslinking factor 1	Prognostic marker in renal cancer (favorable)	Low cancer specificity
20	MKI67	Marker of proliferation Ki-67	Prognostic marker in renal cancer (unfavorable), liver cancer (unfavorable) and pancreatic cancer (unfavorable)	Low cancer specificity
21	MTOR	Mechanistic target of rapamycin kinase	Prognostic marker in renal cancer (favorable)	Low cancer specificity
22	MUC4	Mucin 4, cell surface associated	Gene product is not prognostic	Cancer enhanced (cervical cancer)
23	MUC5AC	Mucin 5AC, oligomeric mucus/gel-forming	Gene product is not prognostic	Group enriched (pancreatic cancer, stomach cancer)
24	MUC6	Mucin 6, oligomeric mucus/gel-forming	Gene product is not prognostic	Group enriched (pancreatic cancer, stomach cancer)
25	MUC16	Mucin 16, cell surface associated	Gene product is not prognostic	Cancer enriched (ovarian cancer)

**Table 3:** Top 10 Ranked MHC Class I Epitopes for CIN3. The top 10 epitopes for the MHC class I molecule play a critical role in activating the adaptive immune system. Rankings were determined using AutoEpiCollect 2.0, which collects data from databases containing immunological data such as the IEDB and VaxiJen. AutoEpiCollect 2.0 predicts T cell eliciting epitope potentials which target any genes of interest by normalizing immunogenicity, antigenicity, and allergenicity data on a 0 to 1 scale. A logarithmic function was applied to the binding affinity for analysis. Epitope potential scores were generated using a machine learning algorithm in AutoEpiCollect 2.0. Ideal epitopes have high immunogenicity and antigenicity values close to 1 and a low allergenicity value close to 0. Results show the pervasiveness of HSPG2 mutations amongst the top 4 epitopes in the CIN3 tissue sample. A total list of epitopes can be found in Supplementary Materials File S5.

Rank	Gene	SNP	Allele	Peptide	Immunogenicity	Antigenicity	Allergenicity	Log(K_d_)	Potential
1	HSPG2	H3087P	HLA-A*30:01	GTRPSNHPT	0.492449	0.614547	0.056338	3.425565	0.655106
2	HSPG2	H3087P	HLA-A*30:01	GTRPSNHPTY	0.507245	0.619365	0.028169	4.098005	0.597095
3	MTOR	V1674G	HLA-A*02:06	KTLGLLLGV	0.596833	0.150391	0.704225	1.128171	0.574449
4	HSPG2	H3087P	HLA-A*30:02	GTRPSNHPTY	0.507245	0.619365	0.028169	4.560696	0.541886
5	MUC16	T2891I	HLA-A*02:03	TLIFEFSEV	0.771128	0.515446	0.788732	1.313724	0.512806
6	TASOR2	R1075P	HLA-A*68:01	NTADEPTTFK	0.752806	0.751365	0.605634	2.170196	0.508856
7	HERC2	S3223A	HLA-B*58:01	LTKAGVVWTW	0.773386	0.236990	0.704225	1.896119	0.507552
8	LRP1	C243S	HLA-B*58:01	FSYANETVSW	0.651741	0.506666	0.732394	1.504077	0.504856
9	MUC16	Y9899F	HLA-A*68:02	TETSAVLFGV	0.605219	0.307394	0.732394	1.517323	0.504358
10	MUC16	T14024P	HLA-B*07:02	IPRLGPYSL	0.537442	0.644582	0.661972	1.795087	0.487651

**Table 4:** Top 10 Ranked MHC Class II Epitopes for CIN3. The top 10 epitopes for the MHC class II molecule. Rankings were determined using the same method as described in Table 3. Results show the pervasiveness of MUC5AC mutations amongst the top 10 epitopes in this CIN3 tissue sample. A total list of epitopes can be found in Supplementary Materials File S6.

Rank	Gene	SNP	Allele	Peptide	Immunogenicity	Antigenicity	Allergenicity	Log(K_d_)	Potential
1	MUC5AC	A684P	HLA-DRB1*01:01	CAPKGVQLGGWRDGV	0.925939	1.000000	0.501253	7.109029	0.747017
2	MUC5AC	A684P	HLA-DQA1*04:01/DQB1*04:02	CAPKGVQLGGWRDGV	0.925939	1.000000	0.501253	7.319653	0.745835
3	MUC5AC	A684P	HLA-DQA1*05:01/DQB1*03:01	CAPKGVQLGGWRDGV	0.925939	1.000000	0.501253	7.461784	0.745035
4	MUC5AC	A684P	HLA-DRB5*01:01	CAPKGVQLGGWRDGV	0.925939	1.000000	0.501253	8.221159	0.740736
5	MUC5AC	A684P	HLA-DRB1*09:01	CAPKGVQLGGWRDGV	0.925939	1.000000	0.501253	8.288741	0.740351
6	MUC5AC	A684P	HLA-DRB4*01:01	CAPKGVQLGGWRDGV	0.925939	1.000000	0.501253	8.330374	0.740114
7	MUC5AC	A684P	HLA-DRB1*15:01	CAPKGVQLGGWRDGV	0.925939	1.000000	0.501253	8.586014	0.738653
8	MUC5AC	A684P	HLA-DRB1*04:05	CAPKGVQLGGWRDGV	0.925939	1.000000	0.501253	8.589704	0.738632
9	MUC5AC	A684P	HLA-DQA1*01:02/DQB1*06:02	CAPKGVQLGGWRDGV	0.925939	1.000000	0.501253	8.704036	0.737977
10	MUC5AC	A684P	HLA-DRB1*07:01	CAPKGVQLGGWRDGV	0.925939	1.000000	0.501253	8.841761	0.737187

**Table 5:** Top 10 Ranked MHC Class I Epitopes for GSM7105215. The top 10 epitopes for the MHC class I molecule play a critical role in activating the adaptive immune system. Rankings were determined using the same method as described in Table 3. Results show the top ranked epitope of BRCA2 amongst the top 50 epitopes in this premenopausal HER2(−)/ER(+)/PR(+) tissue sample. A total list of epitopes can be found in Supplementary Materials File S7.

Rank	Gene	Variant	Allele	Peptide	Immunogenicity	Antigenicity	Allergenicity	Log(K_d_)	Potential
1	BRCA2	V2466A	HLA-A*68:01	QAAAVTFTK	0.823336	0.368606	0.339623	1.870263	0.723582
2	AHNAK2	L4326P	HLA-A*11:01	ASLDVSAPK	0.567767	0.366070	0.509434	1.776646	0.601348
3	AHNAK2	L4321V	HLA-A*68:01	EASVDVSALK	0.560368	0.368136	0.528302	1.715598	0.595889
4	BRCA2	V2466A	HLA-A*68:01	NQAAAVTFTK	0.853711	0.434444	0.509434	2.228939	0.592121
5	AHNAK2	V1133I	HLA-A*68:01	EVDIEAPGAK	0.877111	0.404233	0.207547	3.832114	0.590481
6	AHNAK2	L4326P	HLA-A*68:01	EASLDVSAPK	0.518353	0.391209	0.509434	1.911023	0.576266
7	AHNAK2	M4536L	HLA-A*02:03	KLPEGHLPEV	0.783521	0.435477	0.622642	1.950187	0.544456
8	AHNAK2	P2014S	HLA-A*68:01	EASVDVSAPK	0.560368	0.396187	0.622642	1.704748	0.538244
9	ERBB4	Q98R	HLA-A*31:01	ALNQFRYLR	0.651264	0.746572	0.698113	1.492904	0.516643
10	AHNAK2	L4321V	HLA-A*11:01	ASVDVSALK	0.581320	0.321959	0.603774	2.212660	0.495139

**Table 6:** Top 10 Ranked MHC Class II Epitopes for GSM7105215. Top 10 epitopes for the MHC class II molecule which plays a critical role in activating the adaptive immune system. Rankings were determined using the same method as described in Table 3. Results show the top ranked epitope of AHNAK2 amongst the top 10 epitopes in this premenopausal HER2(−)/ER(+)/PR(+) tissue sample. A total list of epitopes can be found in Supplementary Materials File S8.

Rank	Gene	Variant	Allele	Peptide	Immunogenicity	Antigenicity	Allergenicity	Log(K_d_)	Potential
1	AHNAK2	G5139E	HLA-DQA1*03:01/ DQB1*03:02	LSTEGDSRGCGLEDV	0.938168	1.000000	0.463252	8.215793	0.746206
2	AHNAK2	G5139E	HLA-DQA1*05:01/ DQB1*03:01	DLSTEGDSRGCGLED	0.971488	0.967634	0.492205	8.182241	0.742912
3	AHNAK2	G5139E	HLA-DQA1*03:01/ DQB1*03:02	STEGDSRGCGLEDVP	0.980090	0.934288	0.494432	8.096656	0.734052
4	AHNAK2	G5139E	HLA-DQA1*05:01/ DQB1*03:01	HDLSTEGDSRGCGLE	0.953141	0.885785	0.512249	8.045111	0.707847
5	AHNAK2	G5139E	HLA-DQA1*03:01/ DQB1*03:02	TEGDSRGCGLEDVPV	0.979910	0.818161	0.463252	7.829817	0.694628
6	AHNAK2	M3961V	HLA-DRB1*03:01	KDVTAKDSKFKMPKF	0.746817	0.941983	0.688196	5.173831	0.673084
7	AHNAK2	L2146V	HLA-DRB1*03:01	DVTTKDSKFKMPKFK	0.696015	0.971644	0.634744	5.861925	0.667465
8	AHNAK2	M3961V	HLA-DRB1*03:01	DVTAKDSKFKMPKFK	0.694307	0.971738	0.661470	5.810093	0.665611
9	PIK3CA	C420R	HLA-DRB5*01:01	CSVKGRKGAKEEHRP	0.940360	0.814756	0.766147	8.243493	0.659305
10	AHNAK2	L2146V	HLA-DRB1*03:01	KDVTTKDSKFKMPKF	0.686124	0.942216	0.659243	5.211724	0.655097

**Table 7:** Top MHC Class I and MHC Class II Epitopes for 5 Tumor Samples. Top-ranked MHC Class I and Class II epitopes identified across five tumorigenic samples in case 2. The figure displays the rank 1 epitope for each tumor sample, defined by its source gene and corresponding SNP. Each epitope is paired with the HLA allele showing the highest binding affinity. The “Potential Score” integrates parameters, including binding affinity, antigenicity, allergenicity, and immunogenicity, with higher scores indicating greater therapeutic potential.

	MHC Class I					MHC Class II				
Sample	Gene	SNP	Allele	Epitope	Potential	Gene	SNP	Allele	Epitope	Potential
GSM7105215	BRCA2	V2466A	HLA-A*68:01	QAAAVTFTK	0.72	AHNAK2	G5139E	HLA-DQA1*03:01 HLA-DQB1*03:02	LSTEGDSRGCGLEDV	0.75
GSM7105216	AHNAK2	S1189P	HLA-A*68:01	EASVDVPAPK	0.65	ARID1A	G76R	HLA-DQA1*05:01 HLA-DQB1*03:01	DRAESNGGGGGGGAG	0.74
GSM7105217	BRCA2	V2466A	HLA-A*68:01	QAAAVTFTK	0.74	AHNAK2	G5139E	HLA-DQA1*03:01 HLA-DQB1*03:02	LSTEGDSRGCGLEDV	0.74
GSM7105225	ERBB2	P1209A	HLA-B*07:02	APQPHAPPAF	0.63	AHNAK2	S332A	HLA-DQA1*05:01 HLA-DQB1*03:01	TGAGQGPSSTGQPGR	0.65
GSM7105247	AHNAK2	L4326V	HLA-A*11:01	ASLDVSAPK	0.59	AHNAK2	E616D	HLA-DQA1*05:01 HLA-DQB1*03:01	TEQGREGDATATADR	0.71
GSM7105249	BRCA2	V2466A	HLA-A*68:01	QAAAVTFTK	0.72	AHNAK2	G5139E	HLA-DQA1*03:01 HLA-DQB1*03:02	LSTEGDSRGCGLEDV	0.75

**Table 8:** Sample Profiles of Samples for Analysis. The table illustrates the genetic profile, menopausal status, and therapeutic intervention for all samples analyzed. Tumor samples are matched to non-tumor samples of the same genetic profile to serve as control samples.

Sample	Tissue Type	Menopausal Status	HER2	ER	PR	Therapeutic Intervention
GSM7105215	Tumor	Premenopausal	–	+	+	None
GSM7105216	Normal	Postmenopausal	–	+	+	None
GSM7105217	Tumor	Postmenopausal	–	+	+	None
GSM7105225	Tumor	N/A	N/A	N/A	N/A	N/A
GSM7105247	Tumor	Postmenopausal	–	–	–	None
GSM7105249	Tumor	Premenopausal	–	–	–	None

## Data Availability

The original contributions presented in this study are included in the article/Supplementary Material. Further inquiries can be directed to the corresponding author. AutoEpiCollect 2.0 was developed using Python and is available at https://autoepicollect2.streamlit.app/ (accessed on 2 December 2025). If the webpage is asleep, click the wake up button to reload the page for use. This GitHub repository, https://github.com/sivaGU/AutoEpiCollect-v2.0 (accessed on 2 December 2025), includes the backend code for AutoEpiCollect 2.0. The Supplementary Materials section contains the data collected from this study.

## Supplementary Materials

The following supporting information can be downloaded at: https://zenodo.org/records/17547577 (accessed on 2 December 2025). Supplementary Files S1 and S2. The final annotated output files from Partek Flow for cases 1 and 2, respectively. Supplementary Files S3 and S4. The full list of carcinogenic gene variants for cases 1 and 2, respectively. Supplementary Files S5 and S6. The complete list of top-ranked epitopes for MHC class I and II in case 1. Supplementary Files S7 and S8. The complete list of top-ranked epitopes for MHC class I and II in case 2. Supplementary Files S9. Three-dimensional coordinate PDB files of 3D TCR-pMHC I models for case 1. Supplementary Files S10. Three-dimensional coordinate PDB files of 3D TCR-pMHC II models for case 1. Supplementary Files S11. Three-dimensional coordinate PDB files of 3D TCR-pMHC I models for case 2. Supplementary Files S12. Three-dimensional coordinate PDB files of 3D TCR-pMHC II models for case 2. Supplementary Files S13. Additional information on the need for personalized peptide cancer vaccines for cervical cancer. Supplementary Files S14–S24. PartekFlow RNAseq output files used in the genetic analyses for every sample used in cases 1 and 2.
